# Subinhibitory concentrations of Honokiol reduce α-Hemolysin (Hla) secretion by *Staphylococcus aureus* and the Hla-induced inflammatory response by inactivating the NLRP3 inflammasome

**DOI:** 10.1080/22221751.2019.1617643

**Published:** 2019-05-23

**Authors:** Na Guo, Zuojia Liu, Zhiqiang Yan, Zonghui Liu, Kun Hao, Chuanbo Liu, Jin Wang

**Affiliations:** a State Key Laboratory of Electroanalytical Chemistry, Changchun Institute of Applied Chemistry, Chinese Academy of Sciences, Changchun, People’s Republic of China; b Department of Chemistry and Physics, State University of New York, Stony Brook, NY, USA; c Department of Food Quality and Safety, College of Food Science and Engineering, Jilin University, Changchun, People’s Republic of China

**Keywords:** *Staphylococcus aureus*, α-Hemolysin, Honokiol, inflammasome, binding interaction, molecular docking

## Abstract

*Staphylococcus aureus* (*S. aureus*) is one of the most serious human pathogens. α-Hemolysin (Hla) secreted by *S. aureus* is a key toxin for various infections. We herein report that Honokiol, a natural plant polyphenol, inhibits the secretion and hemolytic activity of staphylococcal Hla with concomitant growth inhibition of *S. aureus* and protection of *S. aureus*-mediated cell injury within subinhibitory concentrations. In parallel, Honokiol attenuates the staphylococcal Hla-induced inflammatory response by inhibiting NLRP3 inflammasome activation *in vitro* and *in vivo*. Consequently, the biologically active forms of the inflammatory cytokines IL-1*β* and IL-18 are reduced significantly in response to Honokiol in mice infected with *S. aureus*. Experimentally, we confirm that Honokiol binds to monomeric Hla with a modest affinity without impairing its oligomerization. Based on molecular docking analyses *in silico*, we make a theoretical discovery that Honokiol is located outside of the triangular region of monomeric Hla. The binding model restricts the function of the residues related to membrane channel formation, which leads to the functional disruption of the assembled membrane channel. This research creates a new paradigm for developing therapeutic agents against staphylococcal Hla-mediated infections.

## Introduction

*Staphylococcus aureus* (*S. aureus*) is one of the most serious human pathogens [1]. *S. aureus* infection is difficult to treat due to its resistance to a wide range of antibiotics [2,3]. The occurrence and nature of *S. aureus* infection critically depend on the various extracellular virulence factors secreted by *S. aureus* [4]. α-Hemolysin (Hla) has emerged as an extracellular toxin secreted by most pathogenic *S. aureu* strains. It is selectively hemolytic and leads to cell damage through initial binding and incorporation into the target cell membrane [5]. The critical roles of Hla in *S. aureus* pathogenesis have been well documented in laboratory animals [6]. Inhibiting the function of Hla therefore provides a paradigm shift to develop a new approach to treating *S. aureus* infections.

NLRs (Nod-like receptors), including NLRP1 (Nucleotide-binding domain and leucine-rich repeat-containing gene family, pyrin domain-containing protein 1), NLRP3 and NLRC4 (NLR family CARD domain-containing protein 4), function as intracellular microbial and nonmicrobial sensors [7]. NLRs associate with NLRP3, ASC (apoptosis-associated speck-like protein containing a caspase recruitment domain), and procaspase-1 to form the NLRP3 inflammasome [8,9]. Subsequently, the NLRP3 inflammasome is activated in response to large amounts of pathogen-derived toxins, such as staphylococcal Hla. The active NLRP3 inflammasome is a signalling complex that secretes the proinflammatory cytokines interleukin (IL) 1*β* and IL-18 and then initiates programmed cellular necrosis. Although purified Hla induces inflammation in mice, rats and rabbits [10–12], the mechanisms by which staphylococcal Hla promotes inflammation in animals remain to be best elucidated. Currently, natural products are receiving increasing interest in the treatment of *S. aureus*-mediated disease. Honokiol (Figure S1(a)), a natural plant polyphenol, has been widely used in medicine for its multiple pharmacological properties [13]. To date, few detailed studies have reported on the effect of Honokiol on staphylococcal Hla.

Examinations of Honokiol in mouse models demonstrated that it protects liver damage caused by *S. aureus* by inhibiting NLRP3 inflammasome activation and the expression of proinflammatory cytokines. Experimentally, we confirmed that Hla is a potential target for Honokiol binding with a modest affinity without impairing its oligomerization. Based on molecular docking analyses *in silico*, we make a theoretical discovery that Honokiol is located outside of the triangular region of monomeric Hla. The binding mode theoretically leads to the functional disruption of the assembled membrane channel by restricting the function of the residues related to membrane channel formation. Our results reveal a new mechanism for developing therapeutic agents against *S. aureus* infections.

## Results

### Honokiol inhibits the production and the hemolytic activity of staphylococcal Hla

**Figure 1.**
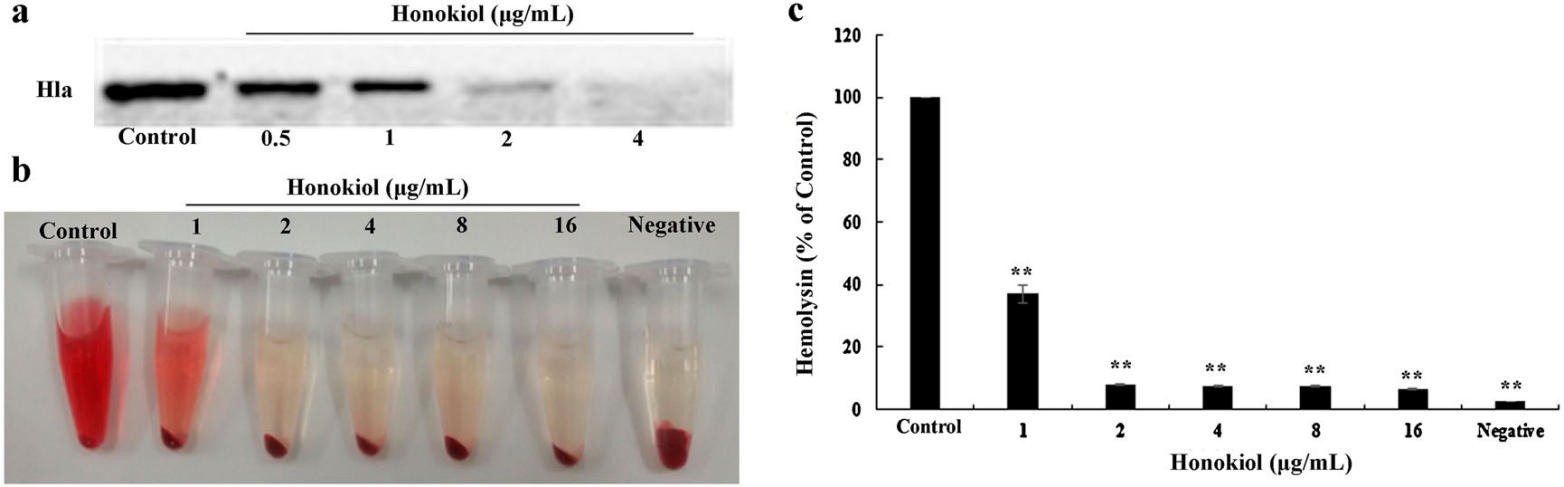
Honokiol inhibits the production and hemolytic activity of staphylococcal Hla. (a) WB analysis of Hla production. Culture supernatants of *S. aureus* 8325-4 grown in the absence or presence of subinhibitory concentrations of Honokiol were detected with a specific antibody against Hla. (b,c) Hemolysis assays were performed with rabbit red blood cells in PBS. The addition of Honokiol reduced the hemolysis, as indicated by the colour attenuation (b), and the OD absorbance at 543 nm decreased (c). Bars show the mean values of the experiments (*n* = 3). **Indicates *P* < 0.01 when compared with the control group.

Our experiments first demonstrated that Honokiol inhibits the growth of *S. aureus* strains *in vitro* and *in vivo* (Figure S1(b and c)). Additionally, the subinhibitory concentrations of Honokiol led to a dose-dependent decrease of Hla secretion from *S. aureus* 8325-4 (Figure 1(a)). Experimentally, the culture in the presence of 2 μg/mL Honokiol led to a recognizable reduction in Hla secretion. During the culture with 4 μg/mL Honokiol, few immunoreactive proteins were detected visibly. In summary, Honokiol directly inhibits Hla production by *S. aureus* 8325-4.

Several lines of evidence have suggested that Hla production by *S. aureus* leads to the hemolysis of rabbit red blood cells (rRBCs), which are highly sensitive to the lytic action of Hla [14,15]. To elucidate the biological relevance of *S. aureus* 8325-4 exposure to Honokiol, a hemolysin release assay was performed (Figure 1(b)). In the absence of Honokiol, *S. aureus* 8325-4 supernatant caused almost complete lysis of rRBCs as indicated by the red colour. Conversely, the addition of Honokiol remarkably protected *S. aureus*-mediated rRBC lysis, as shown by the gradual disappearance of the red colour. In the negative control group, the rRBCs were not lysed by vehicle PBS. The visual colour changes in these assays demonstrate that Honokiol at subinhibitory concentrations prevents staphylococcal Hla-induced lysis of rRBCs. Consistent with the published results [16], this result supports a role for Honokiol in the blockade of the lytic activity of staphylococcal Hla (Figure 1(c)). Together, these observations demonstrate the efficacy of Honokiol as an inhibitor of Hla.

### Honokiol protects A549 cells against *S. aureus*-mediated injury and apoptosis

**Figure 2.**
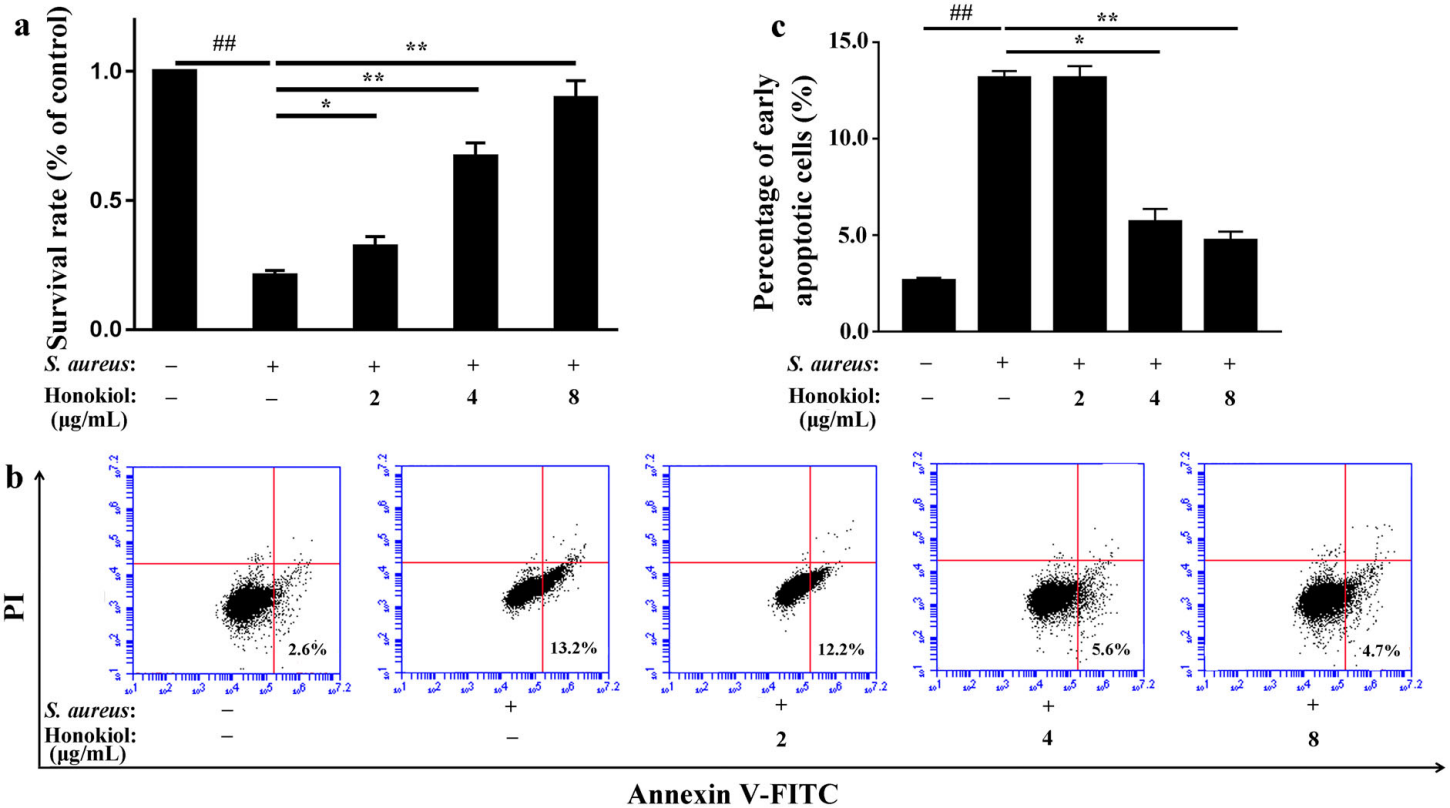
Honokiol protects A549 cells against *S. aureus*-mediated injury and apoptosis. (a) Survival rates of cells treated with *S. aureus* supernatants were compared with the survival rates of the control group treated with PBS in the MTT assay. (b) Apoptosis was analyzed by flow cytometry. A value for early apoptosis (a percentage) is recorded in the lower right-hand corner of each panel. (c) Early apoptotic rates in A549 cells treated with *S. aureus* supernatants. # Represents a significant difference. *Indicates *P* < 0.05 and **indicates *P* < 0.01 when compared with the control group.

Published data have demonstrated that human alveolar epithelial (A549) cell injury and death largely depend on Hla because *S. aureus* strains lacking Hla do not cause cell injury and death [17]. Based on the findings described above, we speculated that Honokiol would protect A549 cells from *S. aureus*-mediated death. The survival rate of A549 cells infected with *S. aureus* 8325-4 decreased to 20.8% compared to that of the uninfected cells (Figure 2(a)). Unexpectedly, the survival rate of the infected cells increased gradually following the addition of Honokiol. At 8 µg/mL of Honokiol, the survival rate of the infected cells even recovered to 89.6%. Additionally, a flow cytometric analysis was performed to determine whether the protective role of Honokiol is associated with cell death. Upon coculture of A549 cells with *S. aureus* 8325-4 in the presence of PBS control, early apoptotic cell death was apparent (Figure 2(b)). However, the addition of 4 and 8 μg/mL Honokiol to the infected cells significantly reduced the number of cells undergoing early apoptotic cell death (Figure 2(c)). These results support the hypothesis that Hla is a major mediator of *S. aureus*-induced cell apoptosis in different cell types [18]. More importantly, our result highlights the potential therapeutic effect of Honokiol on cells exposed to staphylococcal Hla.

### Honokiol inhibits staphylococcal Hla-mediated NLRP3 inflammasome activation

**Figure 3.**
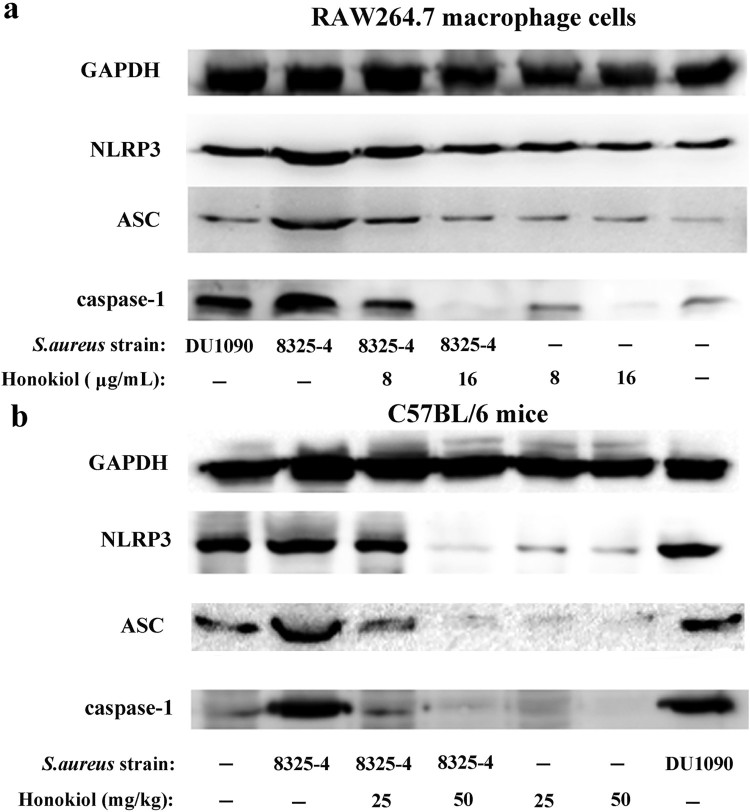
Honokiol inactivates the staphylococcal Hla-induced NLRP3 inflammasome. Immunoblotting analyses of NLRP3, ASC and caspase-1 production by RAW264.7 macrophage cells (a) and by liver tissues from mice (b) exposed to different treatments. GAPDH was used as an internal control.

*S. aureus* can activate the NLRP3 inflammasome in many human cells [7]. In RAW264.7 macrophage cells infected with *S. aureus* 8325-4, the expression levels of the three inflammasome proteins NLRP3, ASC, and caspase-1 were higher than in the same cells infected with DU1090 and PBS (Figure 3(a)). In particular, when compared to the expression of the inflammasome proteins in cells infected with DU1090, the increased expression of the three proteins may be attributed to the Hla secreted by *S. aureus* 8325-4. Following the addition of Honokiol, however, the expression level of the three proteins was gradually attenuated. In contrast, the cells treated with Honokiol in the absence of *S. aureus* 8325-4 showed no change in the expression level of both NLRP3 and ASC proteins. It is particularly noteworthy that a high dose of Honokiol (16 μg/mL) completely inhibited caspase-1 expression. This finding reinforces the notion that high doses of Honokiol induce apoptosis in different cell types [13]. Moreover, the *in vivo* level of NLRP3 inflammasome activation was further examined in mouse liver tissues (Figure 3(b)). In the *S. aureus* DU1090 group, the production of the three proteins was similar to that produced in the control group. Compared to the negative control group, the increased expression of the three proteins indicated that *S. aureus* 8325-4 stimulated their production due to Hla secretion. With the addition of Honokiol, however, the three proteins in the *S. aureus* 8325-4 system were all attenuated, as observed in the cells (Figure 3(a)). Altogether, these results strongly illustrate that Honokiol effectively inactivates the staphylococcal Hla-induced NLRP3 inflammasome *in vitro* and *in vivo*.

### Honokiol suppresses *S. aureus*-induced inflammation in mice

**Figure 4.**
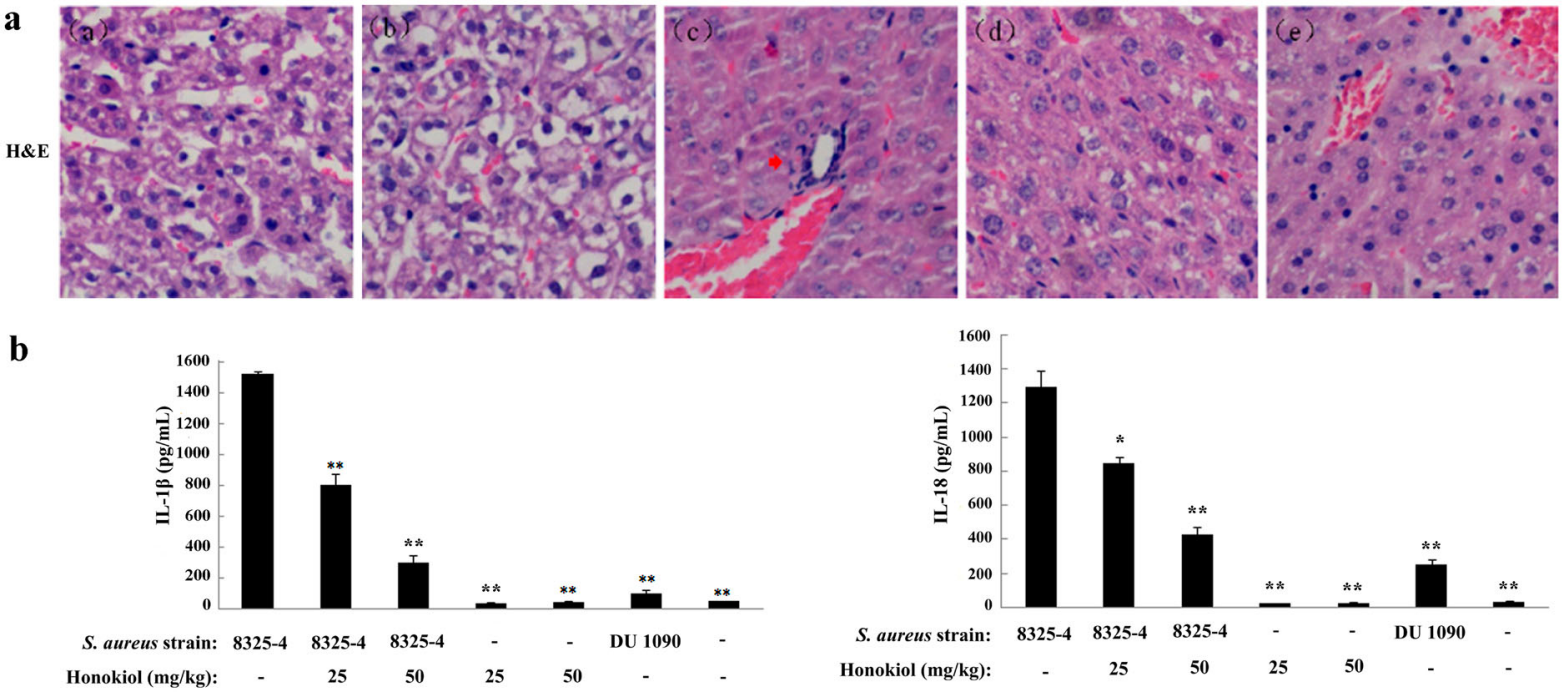
Honokiol suppresses *S. aureus*-induced inflammation in mice. (a) Histological analyses of mouse livers. C57BL/6 mice were administered an intraperitoneal injection of PBS as a control (image a), 200 μL of resuspended *S. aureus* DU1090 as a negative control (image b) or *S. aureus* 8325-4 as a positive control (image c) (1 × 10^8^ CFUs per 200 μL). After 3 h, *S. aureus* 8325-4-infected C57BL/6 mice were treated with 25 and 50 mg/kg of Honokiol (images d and e, respectively) for 24 h. Mouse liver tissues were stained with H&E (×20). The images shown are representative of the results from independent experiments (*n* = 3). The red arrows indicate the infiltrated cells. (b) IL-1*β* and IL-18 production in the blood serum collected from mouse eyes. Mice were treated as described above. *Indicates *P* < 0.05 and **Indicates *P* < 0.01 when compared with the control group.

Accumulating immunohistochemical evidence indicates that Honokiol suppresses inflammation in *S. aureus* 8325-4-infected mice (Figure S2). H&E-stained livers of mice infected with *S. aureus* 8325-4 were examined to assess the protective effect of Honokiol. Few infiltrating inflammatory cells were found in the control mice treated with PBS (image a) and *S. aureus* DU1090 (image b) (Figure 4(a)). However, there was obvious inflammatory cell infiltration (red arrow indicating) in liver tissues of mice infected with *S. aureus* 8325-4 (image c). This observation indicates that the severe infiltration of the inflammatory cells and the damage to mouse livers were caused by staphylococcal Hla. However, it is important to note that the addition of Honokiol significantly alleviated liver damage and fewer infiltrating inflammatory cells were observed (images d and e).

To assess the extent of inflammation, we analyzed the secretion levels of the inflammatory cytokines IL-1*β* and IL-18 (Figure 4(b)). The mice infected with *S. aureus* 8325-4 secreted much higher levels of IL-1*β* than those infected with *S. aureus* DU1090 (left panel). Following treatment with 25 and 50 mg/kg Honokiol, the percentage of secreted IL-1*β* was reduced to 5% and 19%, respectively. Similarly, the mice infected with *S. aureus* 8325-4 secreted marked less IL-18 in a similar manner to the production of IL-1*β* (right panel). Following treatment with Honokiol at the same concentrations indicated above, the percentage of IL-18 was reduced to 67% and 32%, respectively. In contrast, the mice treated with either Honokiol or vehicle produced a relatively small amount of IL-1*β* and IL-18 inflammatory cytokines. These data suggest that Honokiol significantly inhibits the Hla-mediated NLRP3 inflammasome activation.

### Honokiol binds to monomeric Hla without impairing its oligomerization

**Figure 5.**
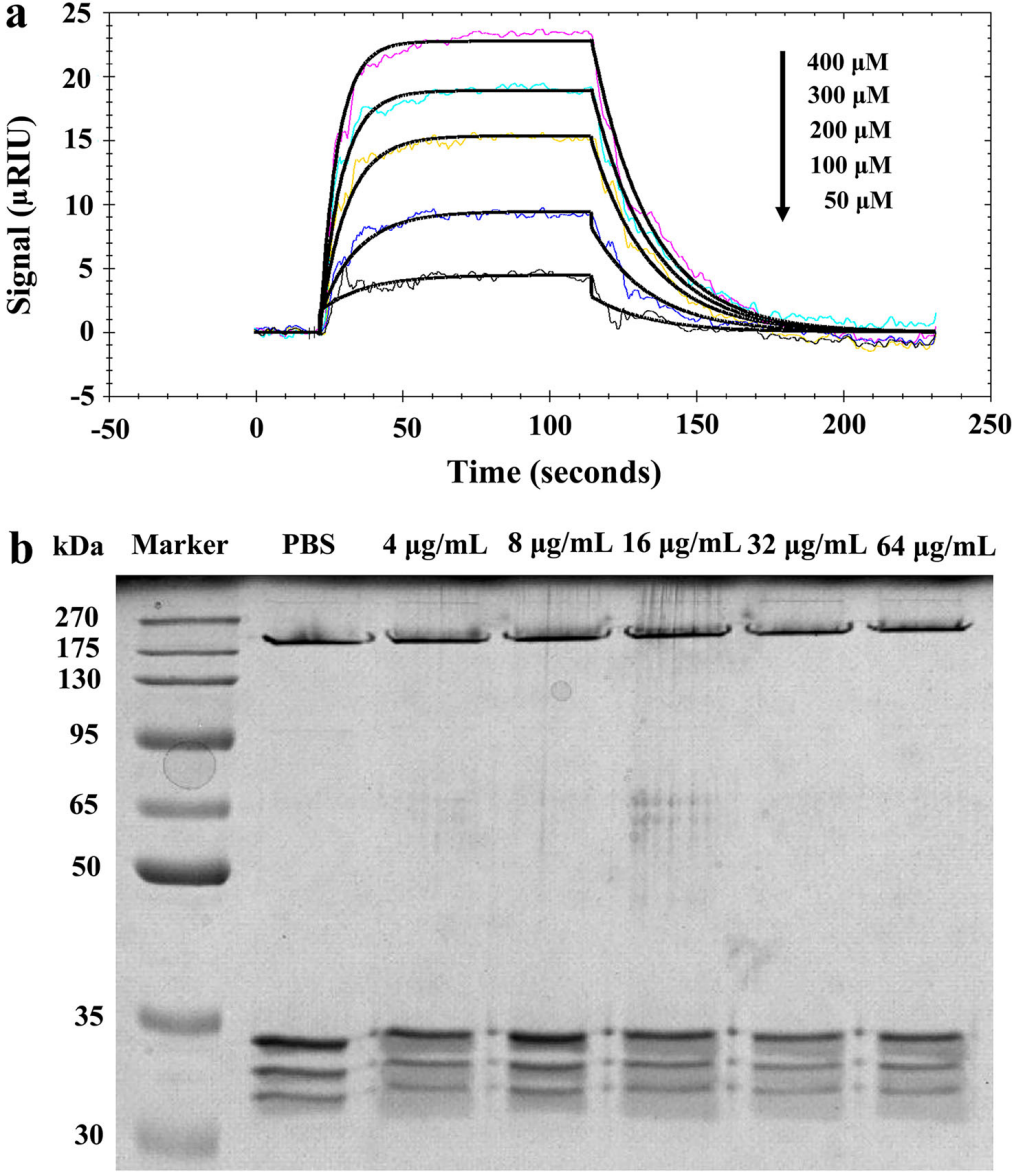
Honokiol binds to monomeric Hla without impairing its oligomerization. (a) The kinetics of Honokiol binding to Hla. The solid black lines show the best fit of the 1:1 binding model. The chromatic lines represent the kinetic signal response to the binding interaction. (b) Honokiol does not disrupt the deoxycholate-induced oligomerization of Hla (designated Hla_7_). The results shown are representative of the results from independent experiments (*n* = 3).

To modulate the biological activity of a given protein, it is a prerequisite for small-molecule drugs to bind to that protein [19]. To better understand the mechanism of action of Honokiol, we experimentally validated its Hla-binding behaviour in SPR assays (Figure 5(a)). The association rate constant Ka (293 M^−1^s^−1^) and the dissociation rate constant Kd (5.03 × 10^−2^ s^−1^) are within the same order of the ligand-protein interactions [19]. The equilibrium dissociation constant (*K_D_* = 1.72 × 10^−4^ M) indicates that Hla might possess an ability to bind Honokiol on the chip surface with a modest affinity relative to the surrounding environment. Previous studies demonstrated that Hla can form a sodium dodecyl sulfate- and heat-stable heptamer within the cell membrane [16,20]. To examine the assembled heptameric state, we tested the effect of Honokiol on Hla oligomerization. When the monomeric Hla (33.2 kDa) was incubated with increasing concentrations of Honokiol, a stable heptamer (Hla_7_, 232.4 kDa) was clearly visualized in a fashion identical to that seen with vehicle PBS (Figure 5(b)). This finding is in good agreement with previous observations in rRBCs treated with purified Hla and *β*-cyclodextrin derivatives [16].

### Computational characterization of Honokiol binding to Hla

**Figure 6.**
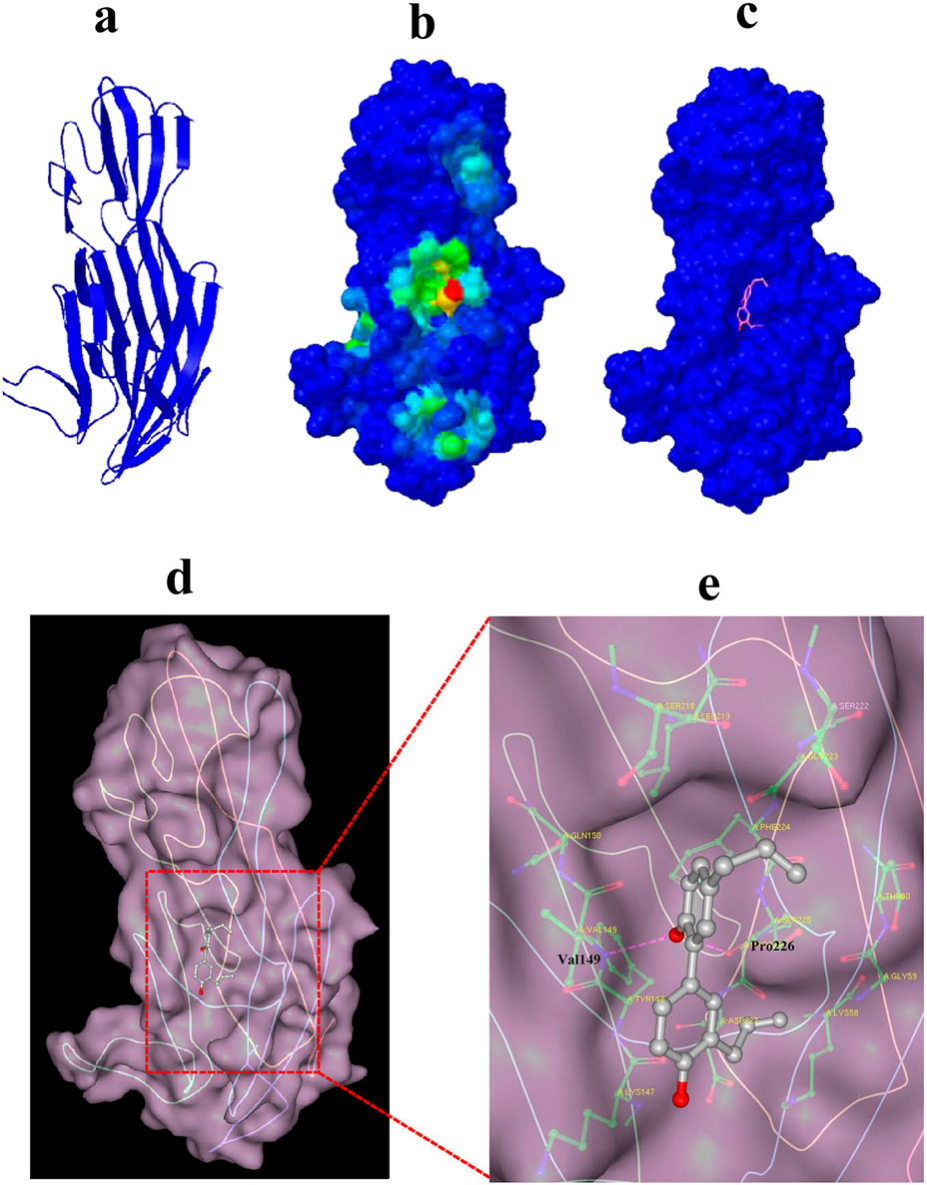
Computational characterization of Honokiol binding to Hla. (a) The monomeric structure of Hla is depicted based on homology modelling. (b) Close-up of the Honokiol docking site (the best energy mode) on the surface of Hla (red dot). Surface presentation demonstrating the structure of Hla (blue). **c** Binding pose of Honokiol in the binding pocket of Hla. (d) The final conformation of Honokiol bound to Hla. (e) The structure of the binding model shows the interaction of Honokiol with Hla residues in the binding pocket. Honokiol moieties are coloured gray and rendered as a stick representation.

To gain insight into the mechanism of the Honokiol-Hla interaction, molecular docking was employed to confirm the preferential binding site and the binding mode. The simulation was based on a monomeric Hla structure obtained from the homology modelling (Figure 6(a)). Cluster analysis displayed the most likely conformation cluster, which corresponded to the most preferred pocket on Hla (Figure 6(b and c)). The binding pocket and the binding pose were further revealed by docking conformation (Figure 6(d)). Honokiol is sealed in the Hla binding pocket and possesses a large contact surface with Hla residues, such as LYS147, TYR148, VAL149, GLN150, PHE224, PRO226, and ASP227 (Figure 6(e)). Furthermore, the computational modelling uncovered that the hydrogen bonds formed between Honokiol and residues VAL149 and PRO226 stabilize Honokiol binding to Hla. This modelled structure characterizes the molecular basis for the biological activity of Honokiol against Hla.

### Predicted Honokiol-Hla binding structure

**Figure 7.**
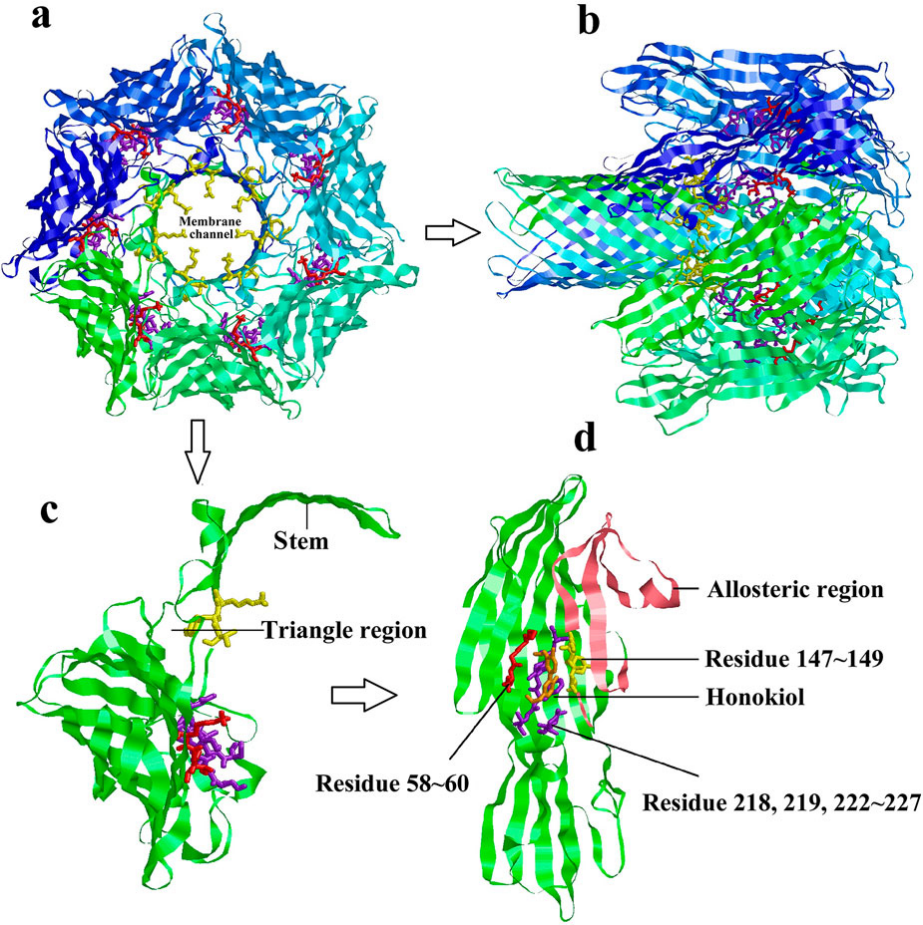
Predicted structure of Honokiol binding to Hla. (a) View of the heptamer down the sevenfold axis (PDB ID 7AHL). Each promoter is shown with a different colour and the predicted residues contacting Honokiol are shown as coloured sticks. (b) Ribbon representation of the heptamer viewed parallel to the sevenfold axis. (c) The ribbon representation of a protomeric subunit structure is extracted from the heptamer with the triangle region and stem labelled. (d) Ribbon representation of monomeric Hla-bound Honokiol with the allosteric region and residue information labelled. The allosteric region is coloured in pink. The residues LYS58, GLY59, and THR60 are coloured red. The residues SER218, LEU219, SER222, GLY223, PHE224, SER225, PRO226, and ASP227 are coloured purple. The residues LYS147, TYR148, VAL149 are coloured yellow. Honokiol is coloured orange. The interacting residues LYS147, TYR148, and VAL149 are located in the structure of the channel. Consequently, Honokiol influences the formation of the membrane channel or the channel structure, which leads to the loss of the heptamer function.

From the view of the heptamer down (Figure 7(a)) or parallel (Figure 7(b)) to the sevenfold axis, it is clear that the LYS147, TYR148, and VAL149 binding residues protrude into the cylinder at the constricted juncture of the triangular region [20]. These residues should play important roles in controlling ion conductance through the membrane channel, which is associated with the hemolytic activity of Hla. As displayed in a protomer subunit structure extracted from the heptamer (Figure 7(c)), the residues LYS147, TYR148, and VAL149 are pulled away from the binding site along with the stem *β*-strands and located outside of the triangle domain. Due to the binding of Honokiol to the pocket within the Hla monomer, these residues could be restricted in the binding pocket and fixed tightly by hydrogen bonding rather than being pulled away from the binding site (Figure 7(d)). As a result, the binding model disrupts membrane channel formation.

## Discussion

*S. aureus* can lead to infection in numerous species, and some secreted virulence factors display high species specificity [21]. Several studies have reported that many natural products show protective effects against *S. aureus* injury by inhibiting the expression or activity of Hla [22,23]. The growth curve assay showed that Honokiol at low subinhibitory concentrations hardly altered the bacterial growth kinetics (Figure S1(b and c)) but inhibited the production of Hla from *S. aureus* 8325-4 (Figure 1). To ensure the protection that Honokiol provides is actually due to its ability to antagonize the toxin and not merely the result of an inhibitory effect on bacterial growth, we quantified its protective effects (Figure 2). These results demonstrate that Hla is a crucial toxin in *S. aureus* injury, but the addition of Honokiol protects A549 cells against *S. aureus*-mediated injury and apoptosis. The low concentration of Honokiol required to elicit this protective effect is very favourable, which highlights the potential for Honokiol as a useful natural product inhibitor of Hla.

The NLRP3 inflammasome is a pattern recognition receptor in the cytoplasm. It can be induced by a variety of environmental irritants, such as bacterial and pore-forming toxins etc [24]. McGilligan et al reported that the NLRP3 inflammasome in rat conjunctival goblet cells can be activated by *S. aureus* [7]. Our results show that the degree of NLRP3 inflammasome activation in response to *S. aureus* 8325-4 was stronger than that to *S. aureus* DU1090 in either the cultured cells or mouse liver tissues (Figure 3 and Figure S2). The results demonstrated that inflammasome activity was mediated by Hla in host cells, this outcome is in agreement with previous studies [25]. NLRP3 inflammasome activation leads to the maturation and secretion of IL-1*β* and IL-18. Their upregulations can be regarded as evidence of inflammation [26]. However, Honokiol inhibits the Hla-induced inflammation, as inferred from the reduced expression of inflammatory proteins in cells and mouse liver tissues (Figure 4). These findings provided compelling evidence that Honokiol inhibits NLRP3-mediated inflammasome activation.

Staphylococcal Hla is a water-soluble monomer that binds to susceptible host cell membranes and subsequently assembles into a stable homoheptameric transmembrane channel [27]. The formation of an oligomeric Hla_7_ channel within the membrane of susceptible cells leads to the outflow of cytoplasmic contents and the inflow of small molecules into the cell as well as cell lysis. Many compounds impair the Hla toxin function by hindering the oligomeric Hla_7_ formation [21]. Compared IB201 has been shown to functionally block ion conductance through the artificial Hla pore [18]. One example study demonstrated the utility of *β*-cyclodextrin lodging in the central pore of monomeric Hla to impair its function [28]. To examine the effect of Honokiol on oligomer formation, we first explored the binding interaction between Honokiol and Hla (Figure 5(a)). The modest binding affinity (*K_D_* = 1.72 × 10^−4^ M) provides direct evidence for the reversible binding of Honokiol to Hla. Compared to the tight binding interaction, Honokiol releases freely from Hla to its surrounding environment. Afterward, Honokiol dissociation functions to inhibit both the production and the hemolytic activity of staphylococcal Hla. Interestingly, the addition of Honokiol to the purified Hla does not prevent the formation of the heptamer (Figure 5(b)). On the basis of the Heptamer structure, two short sections of polypeptide form two sides of the triangle region (PRO103 to THR109 and VAL149 to ASP152), which plays a key role in conformational rearrangements [20]. Honokiol possesses a large contact surface with the Hla VAL149 and PRO226 residues and forms hydrogen bonds (Figure 6). This binding model leads to a theoretical discovery that Honokiol, along with one side of the triangle region, is located on the outside rather than on the inside of the triangle region (Figure 7). This binding model provides a solid basis for disturbing the formation of the heptamer via hindering the monomeric Hla contacts. Furthermore, the inhibitory effect of Honokiol on NLRP3 inflammasome activation does not result from the impaired Hla oligomer formation.

**Figure 8.**
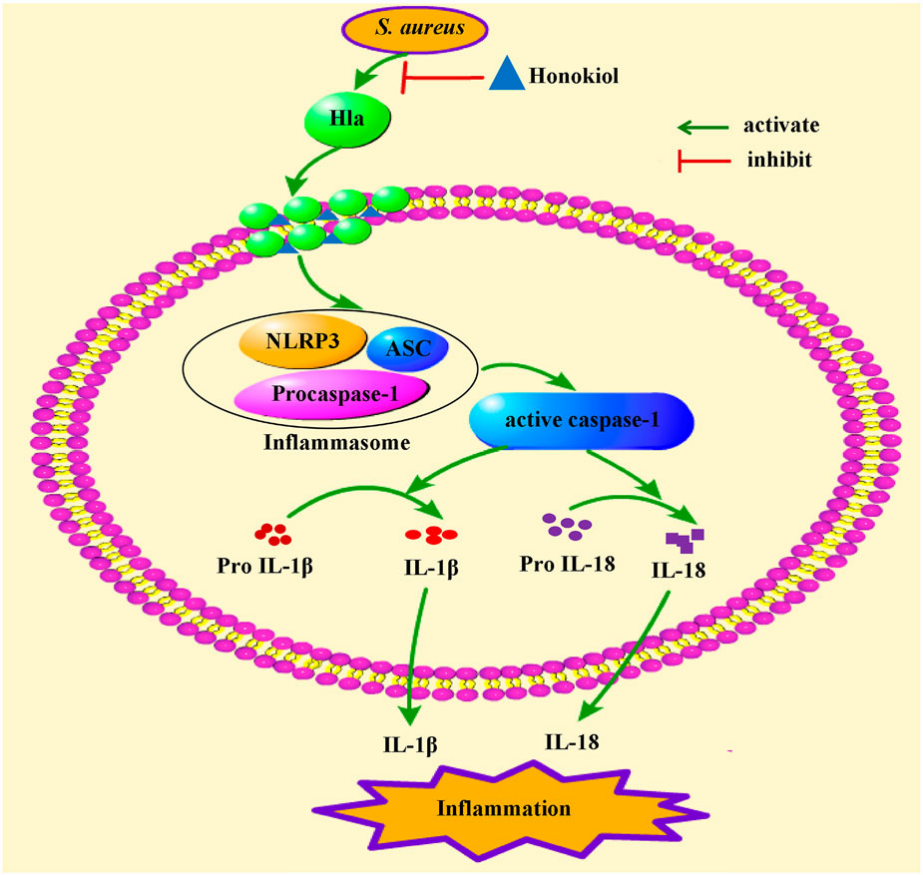
Mechanism of Honokiol-mediated inflammatory response inactivation. On the one hand, Honokiol reduces the production and the hemolytic activity of the staphylococcal Hla directly. On the other hand, Honokiol binds to monomeric Hla without impairing its oligomerization on the membrane of host cells. The resultant Hla-Honokiol complex inhibits the assembly of the NLRP3 inflammasome and the activation of caspase-1, which is involved in the processing of IL-1*β* and IL-18. These events promote apoptotic and pyroptotic cell death in parallel.

In summary, our findings indicate that subinhibitory concentrations of Honokiol attenuate the inflammatory response by inhibiting the production of staphylococcal Hla (Figure 8). Our study proposes that the natural product Honokiol functions as an anti-Hla inhibitor by disrupting the assembled membrane channel rather than impairing heptamer formation. Our results create a new paradigm for developing therapeutic natural products to treat staphylococcal Hla-mediated infections.

## Materials and methods

### Chemical reagents

Honokiol was purchased from the National Institutes for the Control of Pharmaceutical and Biological Products (Beijing, China). For the *in vitro* experiments, Honokiol was dissolved in dimethyl sulfoxide (DMSO, Sigma) under sterile conditions and stored at −20°C. For the *in vivo* experiments, Honokiol was dissolved in phosphate-buffered saline (PBS), and sodium hydroxide was added to adjust the pH to 7.4. Honokiol solution was filtered with a 0.22 μm pore-size acetate syringe filter. Tryptic soy broth (TSB) and Meuller-Hinton broth (MH) were obtained from Difco Laboratories (Detroit, Mich). Dulbecco’s modified Eagle’s medium (DMEM) and fetal bovine serum (FBS) were obtained from Invitrogen (Carlsbad, CA).

### Bacterial strains and cell culture

*S. aureus* 8325-4 and DU1090 (Hla-deficient mutant of 8325-4) strains were gifts from Professor Timothy J. Foster and stored in our laboratory. *S. aureus* DU1090 was constructed by allelic replacement as previously reported [29]. Briefly, plasmid pDU1150 whose *hla* gene was inactivated *in vitro* was cleaved with *Cla*I and ligated with pE194 cleaved with *Taq* I. The *Hla^−^* mutant pDU1346 was constructed by inserting the pE194 *Taq* I fragment between the 0.5 and 0.7 kb *Cla*I fragments which carry *hla*. The shuttle plasmid derivatives of pDU1346, pDU1350 carrying the insertionally inactivated *hla* genes was constructed by ligating *Hind* III-cleaved DNA with an equal amount of *Hind* III-cleaved pCW59 DNA. Then, pDU1350 was transformed into *S. aureus* RN4220, which is capable of stably maintaining chimeric *E.coli-S.aureus* plasmids. The plasmids were isolated from RN4220 and transformed into strain 8325-4. Strains containing the plasmid were selected through replica-plating. Then, the DU1090 strain without hemolytic ability was analyzed and identified as Hla-deficient mutant of 8325-4. Both *S. aureus* strains were grown in TSB overnight until an optical density (OD) at 600 nm was reached. Thereafter, *S. aureus* was diluted 2.5- or 30-fold with DMEM in a conical flask. The diluted *S. aureus* cultures were incubated for 1 h and then treated without and with the indicated concentrations of Honokiol in the *S. aureus* 8325-4 control group and experimental group, respectively. As a negative control, *S. aureus* DU1090 was not treated with Honokiol. After treatment for 16 h, the supernatants were collected and filtered through 0.22 µm pore-size filters. RAW264.7 macrophage cells were purchased from the Chinese Academy of Science Type Culture Collection (Shanghai, China) and cultured in DMEM supplemented with 10% FBS at 37°C in a 5% CO_2_ atmosphere. The cells were plated at 4 × 10^6^ cells/well in 6-well plates for 12 h and then incubated for another 24 h in DMEM without FBS. Then, DMEM media were discarded, and 2 mL of the *S. aureus* supernatants were added to each well. Thereafter, the plate was incubated for 6 h for further analysis.

### Hemolysis assay

The hemolysis activity test of Honokiol was performed according to a previous report [30]. The complete protocol is included in the Supplementary Information.

### MTT assay

Human A549 alveolar epithelial cells, which have been commonly used in biological and physiological studies as a model [23], were employed to evaluate the protective effect of Honokiol against *S. aureus*-mediated injury. A549 cells were seeded into 96-well plates at a density of 5 × 10^3^ cells/well and incubated for 12 h. Each predefined group included five wells. Then, the medium was replaced with 200 μL of *S. aureus* supernatants prepared previously. The viability of cells at 4 h after treatment with *S. aureus* supernatants was evaluated by MTT assay. MTT was added to each well at a concentration of 500 μg/mL. The cells were incubated for an additional 4 h. Thereafter, the medium was discarded, and the crystal violet was dissolved in 150 μL DMSO. The OD_570_ value was recorded using an M200 PRO NanoQuantautoreader (TECAN, Switzerland).

### Qualification of apoptotic cells by flow cytometry

A549 cells were seeded in 6-well plates (2 × 10^5^ cells/well) and cultured overnight. After treatment with *S. aureus* supernatants for 4 h, the cells were detached with trypsin without EDTA and washed in cold PBS. The cells were resuspended in 200 μL binding buffer and incubated with 5 μL of Annexin-FITC and 10 μL of PI for 15 min in the dark. Then, the cells were subjected to flow cytometry (Accuri C6, Ann Arbor, MI).

### Animal experiments

The animal study was approved by “the Institutional Animal Care and Use Committee of Jilin University” (Permit Number: 20160929). Twenty-five male C57BL/6 mice (19–21 g body weight) were obtained from the Experimental Animal Center of Jilin University and randomly divided into five groups. The subsequent protocol is described in the Supplementary Information.

### Immunoblotting assay

After the RAW264.7 macrophage cells were treated and mice were exposed to *S. aureus* 8325-4 or DU1090, proteins were extracted from the cells or liver tissues and subjected to electrophoretic separation. The membranes with transferred proteins were allowed to react with the respective primary antibodies against NLRP3 (Novus Biologicals, 1:1000), ASC (Cell Signaling Technology, Boston, Massachusetts, 1:1000), and caspase-1 (Santa Cruz, Dallas, Texas, 1:100). GAPDH (BD Bioscience, New York, 1:8000) was used as an internal control. The membranes were then incubated with HRP-conjugated anti-mouse or anti-rabbit secondary antibodies (West Grove, PA). Bands were visualized via a chemiluminescence detector (DNR, Kiryat Anavim, Israel).

### Immunohistochemical (IHC) analysis

IHC detection of NLRP3, ASC and caspase-1 proteins was performed with some modifications [12]. The mouse liver tissues were harvested and fixed in 4% paraformaldehyde. Thereafter, these tissues were routinely processed in paraffin blocks and stored in the dark. Then, the fixed tissues were stained with primary antibodies against NLRP3, ASC and caspase-1 or with a hematoxylin and eosin (H&E) kit (Roche). Finally, the samples were scanned with a Carl Zeiss Jena A1 microscope (Carl Zeiss Jena A1, Carl Zeiss Jena Company, Oberkochen, Germany).

### ELISA assay

Blood serum collected from mouse eyes was used to test the expression levels of IL-1*β* and IL-18 by ELISA assay kit following the manufacturer’s protocol.

### Binding interaction measurements

The binding of Honokiol to Hla was measured by a Reichert 4-channel SPR instrument (Reichert). The SPR sensor chip was preconditioned with a 3-minute injection of double-diluted water, and the flow cell surface was activated using 0.05 M NHS/0.2 M EDC for 7 min. Thereafter, the sensor chip was functionalized by immobilizing 0.1 mg/mL Hla on a carboxymethyl dextran chip in 10 mM sodium acetate. The immobilized protein was verified to be approximately 12,000 RU at a flow rate of 25 μL/minute. The unreacted NHS groups were deactivated with 1 M ethanolamine (pH 8.5) for 10 min. Another sensor chip was treated using the same conditions for use as the blank control cell. For binding kinetics analysis, a series of concentrations of Honokiol (50 ∼ 400 μM) were injected. The time interval for each concentration was 90 s binding and 150 s dissociation. The recorded data were subsequently corrected by double referencing to the control sensor chip surface and blank buffer injection signal. Association and dissociation constants (Ka and Kd) were derived from fitting to the concentration-dependent signal responses with a 1:1 kinetic model.

### Oligomerization assay

As described in the deoxycholate-induced oligomerization assay [31], 100 μL of oligomerization mixtures contained 5 mM deoxycholate (Sigma) and 0.5 mg/mL purified Hla. Following the addition of increasing concentrations of Honokiol, the reaction mixtures were incubated at 22°C for 20 min. Thereafter, the mixtures were mixed with 5 × loading buffer and incubated at 37°C for 10 min. Each 25 μL portion of the samples was loaded onto a 10% SDS-PAGE gels for electrophoresis. Gels were stained and then visualized using iBright FL1000 (Thermo Fisher Scientific).

### Molecular docking

Since the binding pocket of Hla for Honokiol was not known in advance, molecular docking experiments were conducted in two steps as described in the Supplementary Information.

### Statistical analysis

Student’s t-test was computed to determine the statistical significance of the results. Differences are statistically significant at *p* < 0.05.

### Data availability

The authors declare that all other data are available within the article and its supplementary information files or available from the corresponding author upon request.

## Supplementary Material

Supplemental Material
