# Supplementary material for: Subinhibitory concentrations of Honokiol reduce α-Hemolysin (Hla) secretion by Staphylococcus aureus and the Hla-induced inflammatory response by inactivating the NLRP3 inflammasome
Source: Emerg Microbes Infect. 2019 May 23;8(1):707–16. doi: 10.1080/22221751.2019.1617643 (PMC6534259; doi:10.1080/22221751.2019.1617643)

**Supplementary Information**

# Subinhibitory Concentrations of Honokiol Reduce α-Hemolysin (Hla) Secretion by *Staphylococcus aureus* and the Hla-Induced Inflammatory Response by Inactivating the NLRP3 Inflammasome

# Na Guo^1,3^, Zuojia Liu^1*^, Zhiqiang Yan^1^, Zonghui Liu^3^, Kun Hao^3^, Chuanbo Liu^1^, and Jin Wang^1,2*^

^1^State Key Laboratory of Electroanalytical Chemistry, Changchun Institute of Applied Chemistry, Chinese Academy of Sciences, Changchun, Jilin, China

^2^Department of Chemistry and Physics, State University of New York, Stony Brook, New York, USA

^3^Department of Food Quality and Safety, College of Food Science and Engineering, Jilin University, Changchun, Jilin, China

# Running title: Honokiol Attenuates Staphylococcal Inflammation

Corresponding authors address: ^1^State Key Laboratory of Electroanalytical Chemistry, Changchun Institute of Applied Chemistry, Chinese Academy of Sciences, 5625 Renmin Street, Changchun, Jilin 130022, P. R. China, Zuojia Liu, Phone: 0086-431-85262003, Fax: 0086-431-85689711, E-mail: [zjliu@ciac.ac.cn](mailto:zjliu@ciac.ac.cn). ^2^Department of Chemistry and Physics, State University of New York, Stony Brook, New York 11794-3400, Jin Wang, Phone: +1-631-632-1185, Fax: +1-631-632-7960, E-mail: [jin.wang.1@stonybrook.edu](mailto:jin.wang.1@stonybrook.edu).

**Supplementary methods**

**Growth curve of *S. aureus* strains**

The bactericidal kinetics were tested by inoculating MH-containing 1/4 × minimum inhibitory concentrations (MICs), 1/2 × MICs and 1 × MICs of Honokiol (dissolved in DMSO) to an initial inoculum with OD_600_ of 0.3. The final DMSO concentration for all conditions was 1% (v/v). A control culture received only 1% DMSO. Following the addition of Honokiol (or 1% DMSO) into 20 mL MH, bacteria were cultured at 37ºC with aeration and cell growth was monitored spectrophotometrically by measuring OD_600_ at each time point (0, 1, 3, 6, 9, 12 and 24 hour(s)).

**Antimicrobial susceptibility test**

MICs of Honokiol against both the *S. aureus* strains were determined using the broth microdilution method following the Clinical and Laboratory Standards Institute guidelines^1^. In short, the overnight grown *S. aureus* strains in TSB were diluted with MH to obtain a concentration of 10^5^ colony forming units (CFUs) per mL. Then, 100 µL *S. aureus* strains suspended in MH were inoculated into 96-well plate. Honokiol was diluted with MH, and 2-fold serial dilutions (100 µL) were added into each well of 96-well plate. Wells without Honokiol treatment served as positive control, while wells containing MH served as negative control. Thereafter, the plate was incubated at 37ºC for 24 hours. The MICs were defined as the lowest concentration of antibiotic which produced complete inhibition of visible growth.

**Hemolysis assay**

Briefly, *S. aureus* were incubated in TSB containing the graded subinhibitory concentrations of Honokiol to the early stationary growth phase (OD_600_ = 2.5). (1) *S. aureus* solutions were centrifuged (5500×g, 4ºC, 1 minute), and then 100 µL supernatants were transferred to 875 µL PBS buffer. Simultaneously, 100 µL medium served as the negative control, and the supernatants without Honokiol treatment served as the positive control (100% hemolysis). Then, the mixture of *S. aureus* supernatants and PBS were incubated with 25 µL defibrinated rabbit blood at 37ºC for 30 minutes and the whole samples were centrifuged (5500×g, 4ºC, 1 minute). Photos were taken by a camera (Nikon D7100) for the qualitative analysis. The OD_543_ of the supernatants was measured by a spectrophotometer (Philes, Nanjing, China) for the quantitative analysis. The percentage of hemolysis was determined based on a comparison with the positive control. (2) The culture sample collected from the post-exponential growth-phase cultures mentioned above were used for the Western blotting (WB) assay. In brief, the equal volumes of culture supernatants (28 µL) were subjected to sodium dodecyl sulphate-polyacrylamide 12% gel electrophoresis at 120 V and then transferred into polyvinylidene fluoride (PVDF) membranes (Upstate, Millipore). Thereafter, the PVDF membranes were incubated with the primary antibody against Hla and HRP-conjugated goat anti-rabbit antiserum (Sigma) as the secondary antibody. Finally, the reacted patterns were visualized with ECL substrate (Beyotime, China), and the images were obtained using a CanoScan LiDE 100 scanner (Canon, Japan). Protein blots were measured using the Image-J software in order to obtain the accurate results.

**Animal experiments**

Mice were kept on a 12-hour light/dark cycle and housed live to a cage with food and water available ad libitum. *S. aureus* strains 8325-4 and DU1090 were grown at 37ºC in TSB overnight and then resuspended in PBS to an OD_600_ of 0.4. Three experimental groups of mice were administrated by intraperitoneal injection of 200 µL resuspended *S. aureus* 8325-4. To investigate the effect of Honokiol, two groups of the three experimental groups of mice were administered 100 µL of Honokiol (25 or 50 mg/kg of body weight) subcutaneously 3 hours after infection with *S. aureus* 8325-4 and then at 12 hours intervals. The third group of mice was treated with 100 µL of sterile PBS as the control group on the same schedule. The fourth group of mice was infected with 200 µL resuspended *S. aureus* DU1090. As a control, this group of mice was treated with 100 µL of sterile PBS on the same schedule. The fifth group of mice served as negative control with injection of 200 µL PBS. After treatment 12 hours, all mice were sacrificed and blood was sampled from eyes. Blood serum was obtained for ELISA analysis. Livers were weighed and homogenized for the calculation of bacteria burden by the serial dilution and plating method. For histopathologic and immunoblotting analysis, the livers were placed in 4% paraformaldehyde (Sigma) and stored at -80ºC, respectively.

**Structural modeling of the monomeric Hla**

To date, the structure of the wild-type monomeric Hla hasn't been determined experimentally though the crystal structure of Hla heptamers was reported^2^. In this work, the wild-type monomeric structure of Hla was modeled by homology modeling. The sequence of Hla was taken from the PDB 7AHL. The sequence alignment and homology modeling were carried out through the web server SWISSMODEL. The monomeric structure (PDB ID 4YHD, chain A, residue 5~293) with the highest sequence identity 99.32% was taken as the template to model the wild-type monomeric Hla. The modeled structure was taken as the docking target for Honokiol.

**Molecular docking**

The docking between Honokiol and Hla was performed with the software AutoDock4.2. Since the binding pocket of Hla for Honokiol is not known in advance, molecular dockings were conducted in two steps, the blind docking and the fine docking. First, in order to seek the preferred binding site of Honokiol onto the surface of Hla, the blind docking was performed by searching for the whole surface of Hla. The docking box was set to be enough large to encompass Hla with the grid numbers of 120×120×120 and grid spacing of 0.50 Å. Fully informed particle swarm algorithm was used to optimize the ligand pose by minimizing the binding energy. 500,000 moves were evaluated for each docking run so that the searching is converged to an energy minimum^3^. 1,000 separate docking runs were conducted between Honokiol and Hla. With the coordinates of 1,000 docked complex conformations, the contacts between the atoms of Hla and Honokiol can be extracted. A contact was defined when the distance between the atoms is below a cutoff distance (5.0 Å). Contact probability of each atom interacting with Honokiol was computed for Hla. In this way, the binding pocket can be located on the surface of Hla. Second, the fine docking was performed around the binding pocket with a smaller binding box (grid numbers 120×120×120 and grid spacing 0.375 Å). The binding pose with the lowest binding energy was picked up for further analysis.

**References**

1. Wikler, M. A. *et al*. Methods for Dilution Antimicrobial Susceptibility Tests for Bacteria That Grow Aerobically; Approved Standard-Eighth Edition. (Clinical and Laboratory Standards Institute. Press, 2009).

2. Sully, E. K. *et al*. Selective chemical inhibition of agr quorum sensing in *Staphylococcus aureus* promotes host defense with minimal impact on resistance. *PLoS Pathog* **10**, e1004174 (2014).

3. Guo, L. Y. *et al*. A comparison of various optimization algorithms of protein-ligand docking programs by fitness accuracy. *J Mol Model* **20**, 2251 (2014).

**Supplementary results**

**Honokiol inhibits the survival growth of *S. aureus* strains**

The minimum inhibitory concentrations (MICs) of Honokiol against *S. aureus* 8325-4 and DU1090 strains were 32 μg/mL. This implies that Honokiol can be an important compound for the development of an anti-*S. aureus* drug. The growth curves of both the *S. aureus* strains cultured with the graded subinhibitory concentrations of Honokiol were shown in Fig. S1b. Honokiol at the level of 1/4 × MICs had little effect on the growth of *S. aureus* 8325-4 (top panel). However, during culture with 1/2 × MICs of Honokiol, the growth rate was significantly decreased. After 6, 9 and 12 hours of Honokiol treatment, OD_600_ value was 30.0%, 28.0% and 28.3% of the control culture, respectively. At the concentration of 1 × MICs, Honokiol completely inhibited the growth of *S. aureus* 8325-4. The growth of *S. aureus* DU1090 was influenced under the identical conditions and the similar behaviors of growth were illustrated (bottom panel).

Based on these *in vitro* findings, we further investigated the inhibitory effect of Honokiol *in vivo*. An established murine liver damage model was employed using viable *S. aureus*. The bacterial burden was quantified to evaluate the inhibition efficacy of Honokiol on *S. aureus* strains survival within the mouse liver tissues. As shown in Fig. S1c, after 24 hours culture, the colony forming units (CFUs) of *S. aureus* postinfections from mice treated with Honokiol were significantly lower than the control group treated with PBS. In the experimental group treated with *S. aureus* 8325-4 (top panel), the CFUs containing 25 and 50 mg/kg of Honokiol was about 82% and 64% of the CFUs in mouse liver tissues treated by PBS, respectively. Notably, the amount of CFUs in liver tissues in mice challenged with *S. aureus* strain DU1090 lacking Hla expression was indistinguishable from that seen in mice treated with *S. aureus* strain 8325-4 (bottom panel). However, PBS as negative control did not directly lead to any colony formations. Combined with the data, we conclude that Honokiol acts as an inhibition on the survival growth of *S. aureus* strains in liver tissues of mice.

**Honokiol** **suppresses the** ***S. aureus* inflammation in mice**

The distribution of the NLRP3, ASC, and caspase-1 proteins, as determined by IHC analysis, varied greatly among the liver tissues from different groups (Fig. S2). The expressions of the NLRP3, ASC, and caspase-1 proteins were few in the control samples. In contrast to the controlled mice, these proteins were observed in liver tissues in mice challenged with live *S. aureus* 8325-4. Although liver tissues in mice challenged with Hla-deficient live *S. aureus* DU1090 treatment were also positive for these proteins, the expression levels were significantly lower than those in *S. aureus* 8325-4 treatment mice. These results suggest that the enhanced expression levels are correlated with the extra production of Hla by *S. aureus* 8325-4. Among the *S. aureus* 8325-4 treatment mice, the three proteins were reduced in the mice treated with Honokiol. Accumulating IHC evidence implies that Honokiol suppresses the inflammation in *S. aureus* 8325-4 treated mice by reducing the secretion of Hla.

**Figure S1. Honokiol inhibits the survival growth of *S. aureus* strains**. **a** Chemical structure of Honokiol. **b** Growth curve for *S. aureus* 8325-4 (top panel) and DU1090 (bottom panel). Values are the average of the results from independent experiments (*n* = 3). **c** Bacterial burden in the livers of the infected mice 24 hours postinfection. ^*^ indicates *P* < 0.05 and ^**^ indicates *P* < 0.01 when compared with the control group.


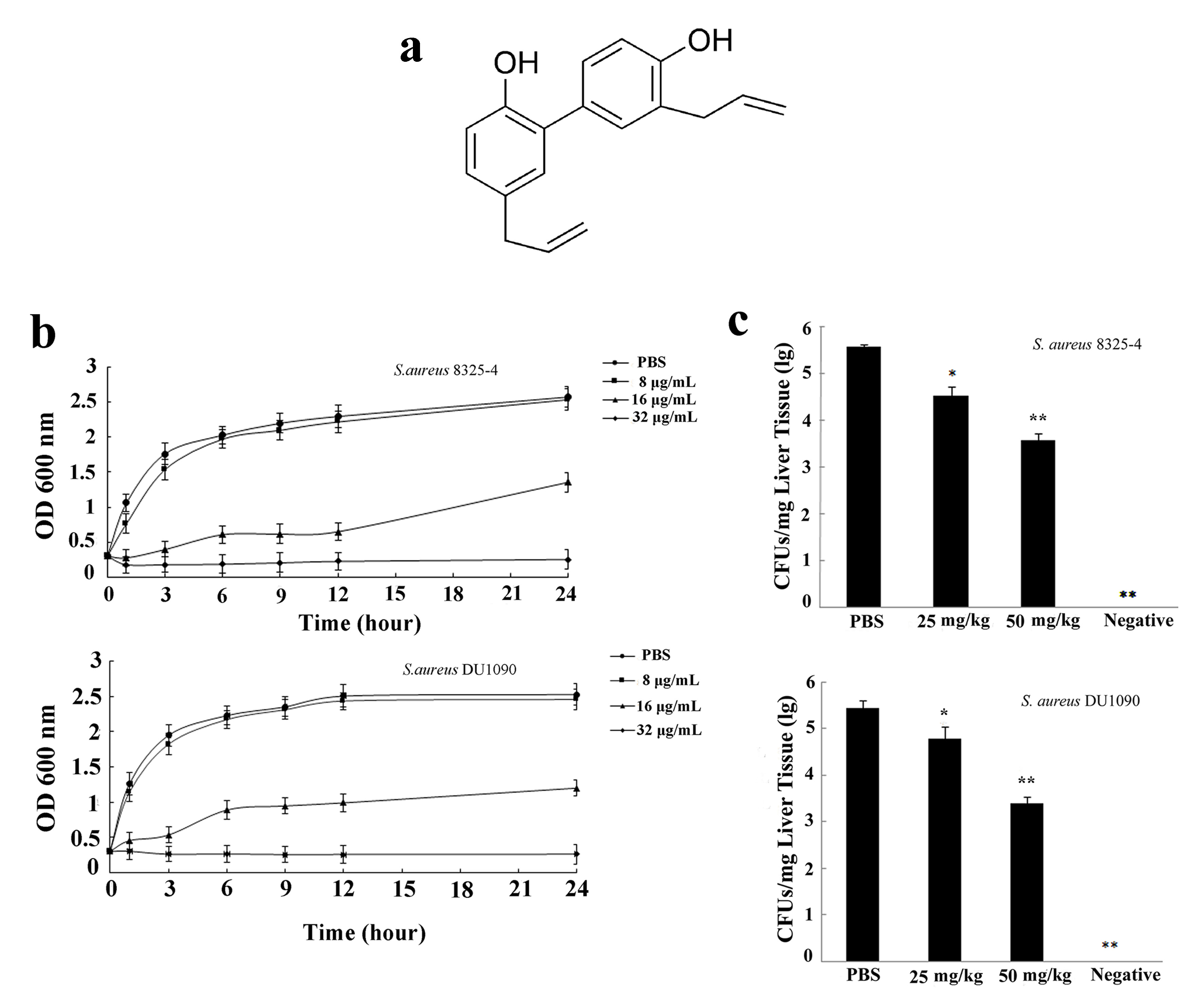


**Figure S2. IHC detection of the NLRP3, ASC and caspase-1 proteins in mouse livers.** C57BL/6 mice were administrated with an intraperitoneal injection of PBS as control (image a) and of 200 μL resuspended *S. aureus* DU1090 as negative control (image b) or *S. aureus* 8325-4 as positive control (image c) (1×10^8^ CFUs per 200 μL). After 3 hours treatment with *S. aureus* 8325-4, C57BL/6 mice were treated with 25 and 50 mg/kg of Honokiol (images d and e) for 24 hours, respectively. Respective primary antibody against the NLRP3, ASC and caspase-1 proteins was used for IHC analysis. Images shown were representative of the results from independent experiments (*n* = 3). The red arrows indicate the NLRP3, ASC and caspase-1 inflammasome expressed in mouse livers.


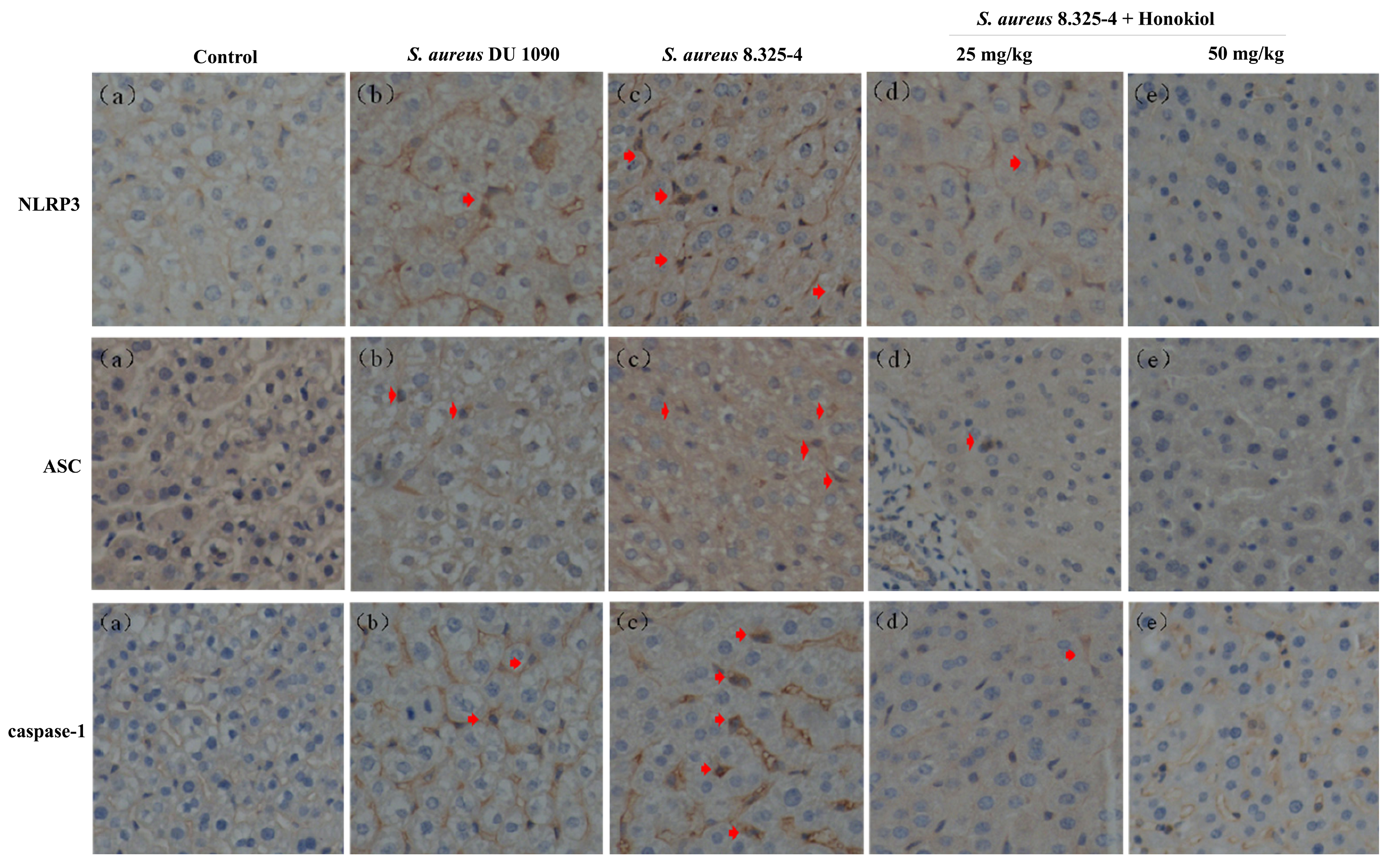

Supplement: Supplemental Material [file TEMI_A_1617643_SM4221.doc]
